# Disulfiram suppressed ethanol promoted RANKL-induced osteoclastogenesis in vitro and ethanol-induced osteoporosis in vivo via ALDH1A1-NFATc1 axis

**DOI:** 10.18632/aging.102279

**Published:** 2019-10-08

**Authors:** Yewei Jia, Jiawei Jiang, Kangxian Zhao, Tan Zhang, Peng Sun, Jiaxuan Peng, Qichang Yang, Yu Qian

**Affiliations:** 1 Department of Orthopaedics, Shaoxing People’s Hospital (Shaoxing Hospital, Zhejiang University School of Medicine, Shaoxing), Zhejiang 312000, China; 2 Department of Orthopaedics, Sir Run Run Shaw Hospital, School of Medicine, Zhejiang University, Hangzhou, Zhejiang 312000, China; 3 Department of Urinary Surgery, Jinhua Central Hospital (Jinhua Hospital, Zhejiang University School of Medicine, Jinhua), Zhejiang 321000, China; 4 Guangxi Key Laboratory of Regenerative Medicine, Guangxi Medical University, Guangxi 530021, China

**Keywords:** disulfiram, ethanol, ALDH1A1, NFATc1, osteoclastogenesis

## Abstract

Excessive alcohol consumption is positively related to osteoporosis, and its treatment strategies are poorly developed. Disulfiram inhibits receptor activator of nuclear factor kappa-B ligand (RANKL)-induced osteoclastogenesis; however, whether it can be used for ethanol-induced osteoclastogenesis and its underlying mechanism are still unclear. In this study, we demonstrated that ethanol promoted RANKL-induced osteoclast formation and bone resorption, whereas, disulfiram suppressed ethanol-induced osteoclastogenesis by abrogating the expression of nuclear factor of activated T cell c1 (NFATc1) in vitro. Further analysis revealed that aldehyde dehydrogenase 1A1 (ALDH1A1) is important for the expression of NFATc1, the master regulator of osteoclast differentiation. Furthermore, we showed that disulfiram protected ethanol-induced osteoporosis in vivo. Overall, our study provides promising evidence that disulfiram can be used as a treatment strategy for alcohol-related osteoporosis via the ALDH1A1T–NFATc1 axis.

## INTRODUCTION

Alcohol is widely consumed worldwide and alcohol use is a leading risk factor for various diseases, accounting for nearly 10% of deaths among populations aged 15–49 years globally. Moreover, alcohol consumption may have negative consequences for the health of future populations because of the current lack of policy action [1]. Accumulated evidence has confirmed that excessive alcohol consumption results in various diseases [2–5]. such as bone diseases. Chronic alcohol intake is a contributing factor to the development of osteoporosis [6, 7]. Although alcohol-related osteoporosis is considered a severe disease, the therapy strategy remains poor.

Osteoporosis is a multifactorial disease that results from dysregulation of bone metabolism or homeostasis. Bone density and integrity are maintained through complex networks and numerous interactions between different bone cell types and their environment, which ensures that bone remodeling is balanced during growth and adulthood [8–10]. Osteoclasts (OCs) represent the only known cell type capable of resorbing the bone matrix, and excessive bone resorption by OCs leads to osteoporosis and tumor-associated bone destruction [11], while impaired OC formation and/or bone resorption result in osteoporosis [12]. A previous study showed that ethanol stimulation increased OC-induced bone resorption areas in a dose-dependent manner [13]. In osteoblasts, ethanol-generated reactive oxygen species increased the expression of receptor activator of nuclear factor kappa-B ligand (RANKL) [14].

Disulfiram, an anti-alcoholism drug, has long been clinically used for the treatment of chronic alcoholism, and previous research revealed its inhibition on RANKL-induced osteoclastogenesis [15]. However, the effect of disulfiram on alcohol-related osteoporosis is still unclear. We thus further explored whether disulfiram owned its protective effect on alcohol-related osteoporosis and its underlying mechanism.

Ethanol is first metabolized into acetaldehyde through several enzymatic and nonenzymatic mechanisms [16], and acetaldehyde has been suggested to contribute to alcohol abuse and alcoholism. Thus, we focused on the role of acetaldehyde dehydrogenase (ALDH) in ethanol-induced osteoporosis. Humans contain multiple isoforms of ALDH, which are divided into nine major families [17]. However, only some of these isoforms are significantly involved in acetaldehyde metabolism. An important role in acetaldehyde oxidation has only been demonstrated for ALDH1A1, ALDH1B1, and ALDH2 [18]. ALDH1A1 is a highly abundant cytosolic protein found in the human liver [19, 20], and accumulated evidence has shown that ALDH1A1 is related to alcohol-induced flushing, as well as alcohol sensitivity and dependence [21, 22]. Disulfiram was previous performed an effective inhibitor which targeted at ALDH1A1, dramatically downregulated the expression of ALDH1A1 in breast cancer [23]. The ALDH1A1 was thus investigated during the process of ethanol-induced osteoclasogenesis.

In the present study, we first demonstrated disulfiram suppressed ethanol-induced osteoporosis either in vitro or in vivo, the deep mechanism contributed to the ALDH1A1-NFATc1 axis.

## RESULTS

### Disulfiram abrogated ethanol-induced OC formation and bone resorption *in vitro*

**Figure 1:**
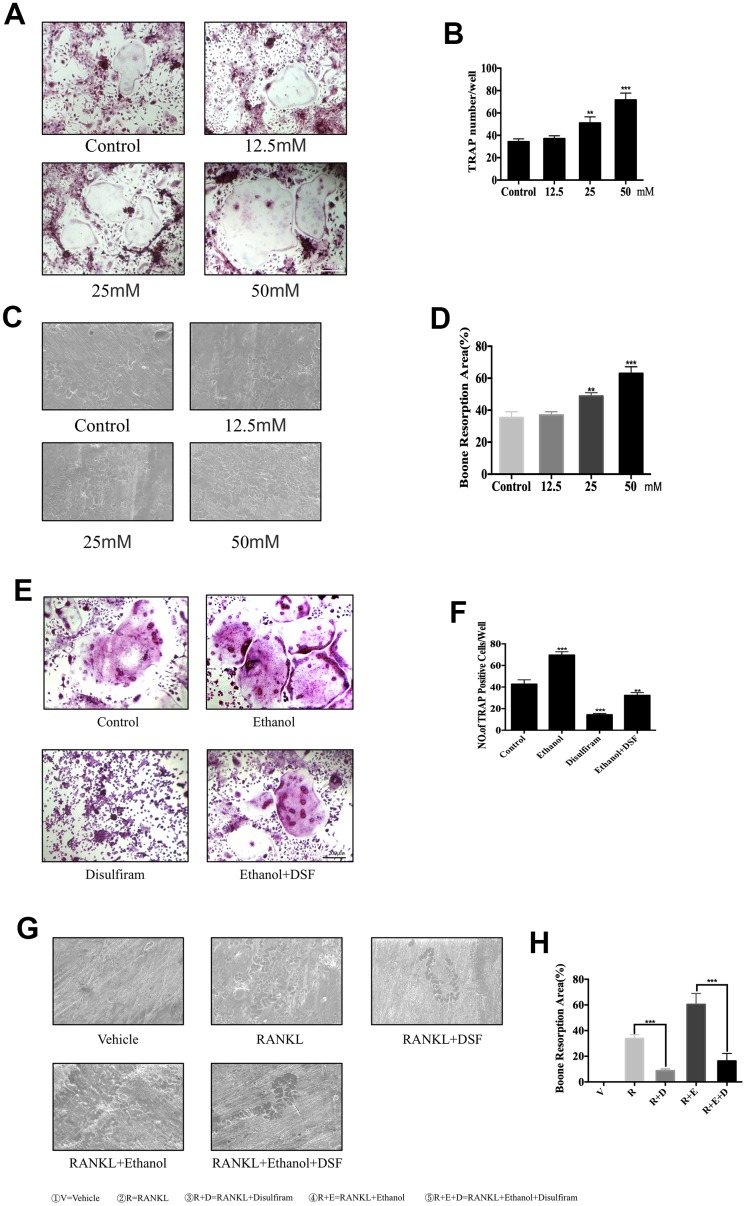
**Ethanol increases RANKL-induced OC formation and bone resorption *in vitro*.** (**A**) BMMs were stimulated with RANKL at different concentrations of ethanol and histochemically stained for TRAP detection. TRAP-positive cells with ≥3 nuclei were considered as OCs. (**B**) Numbers and areas of TRAP-positive multinucleated (>3 nuclei) cells formed in the presence of increasing concentrations of ethanol (*n* = 3). (**C**) Equal number of pro-osteoclasts were cultured on bone slices in the presence of different concentrations of ethanol. After 5 days, bone resorption lacunae were observed by scanning electron microscopy. (**D**) Area of bone resorption was measured using ImageJ software. (**E**) Disulfiram dramatically inhibited RANKL-induced osteoclastogenesis. TRAP-positive cells with ≥3 nuclei were considered OCs (magnification 100×; scale bar = 200 μm). (**F**) Analysis of the numbers and areas of TRAP-positive multinucleated (>3 nuclei) cells (*n* = 3). (**G**) Equal number of pro-osteoclasts were cultured on bone slices treated with indicated condition. After 5 days, bone resorption lacunae were observed by scanning electron microscopy. (**H**) Area of bone resorption was measured using ImageJ software. Data are the mean ± SD. *p < 0.05, **p < 0.01, and ***p < 0.001 compared to the respective controls.

BMMs were cultured with 100 ng/mL RANKL and 25 ng/mL M-CSF for 7 days, with or without 12.5, 25, or 50 mM ethanol, and fixed and stained at the end of experiment to visualize TRAP activity. The data indicated that ethanol significantly increased the cell size and number of TRAP-positive multinucleated cells in a concentration-dependent manner compared to those observed without ethanol treatment (Figure 1A). The numbers and areas of TRAP-positive multinucleated (>3 nuclei) cells were analyzed and the results are shown in Figure 1B.

Because OCs represent the only cell type with bone resorption function, we further investigated the effect of ethanol on bone resorption. BMMs were seeded onto bovine cortical bone slices without or with different concentrations of ethanol, and ethanol concentration-dependently increased the total resorption area (Figure 1C and 1D).

Later, 100nM disulfiram was used to explore its inhibition on ethanol-induced osteoclastogenesis. Our data indicate that disulfiram dramatically inhibited the size and number of TRAP-positive multinucleated cells, which were stimulated by ethanol, compared to those in the RANKL-treated group (Figure 1E and 1F). Bone resorption also revealed the same effect (Figure 1G and 1H).

These data demonstrate that disulfiram dramatically abrogated ethanol-induced OC formation and bone resorption.

### Disulfiram suppressed osteoclast-related genes expression

**Figure 2:**
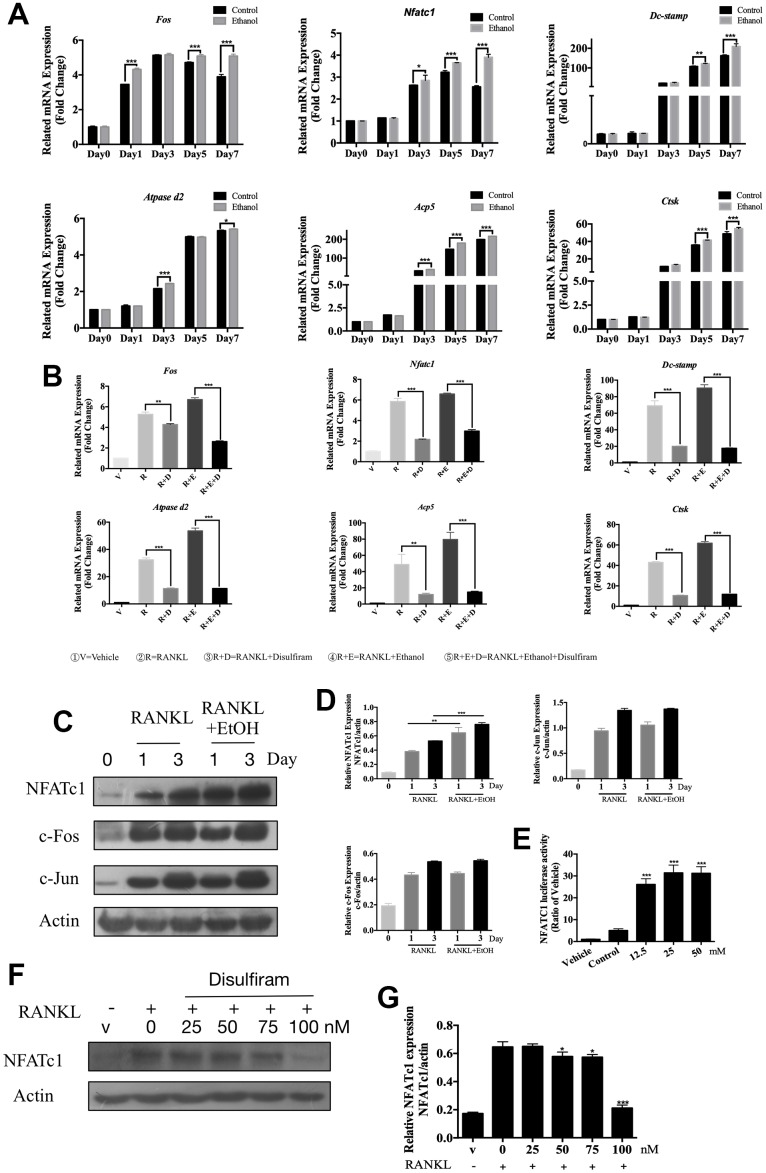
**Disulfiram suppressed osteoclast-related genes expression and down-regulated the expression of NFATc1.** (**A**) qPCR was used to measure relative expression levels, normalized to that of β-actin, of RANKL-induced OC-related genes at different times of treatment with 50 mM ethanol (*n* = 3). (**B**) qPCR was used to measure relative expression levels, normalized to that of β-actin, BMMs were treated with indicated conditions indicated below figure 2B for 3 days (*n* = 3). (**C**) Ethanol increased the RANKL-induced NFATc1 protein expression but did not affect c-Fos and c-Jun. Total cellular proteins were extracted from BMM-derived OCs co-treated with RANKL and 50 mM ethanol for 0, 1, and 3 days. (**D**) Relative expression of c-Fos, c-Jun, and NFATc1 was determined by densitometric analysis of each band and expressed as a ratio to that of β-actin using ImageJ. (**E**) Ethanol stimulated NFATc1 transcriptional activity. RAW 264.7 cells stably expressing the NFATc1-TA-Luc luciferase reporter were pretreated with 50 mM ethanol for 1 h and then stimulated for 6 h with RANKL, after which luciferase activity was measured. Results are expressed as fold-changes compared to the levels in unstimulated controls (*n* = 3). (**F**) Disulfiram inhibited the expression of NFATc1 in a dose-dependent manner. (**G**) Relative expression of NFATc1 was determined by densitometric analysis of each band and expressed as a ratio to that of β-actin using ImageJ software. Bar graphs are presented as the mean ± SD. *p < 0.05, **p < 0.01, and ***p < 0.001.

The qPCR assay was conducted to determine the expression levels of genes involved in OC formation and bone resorption (Table 1). The data showed that ethanol significantly upregulated genes involved in OC formation, such as *c-Fos* and *Nfatc1*, as well as genes involved in precursor cell fusion, such as those encoding dendritic cell-specific transmembrane protein (Dc-stamp) and ATPase H^+^ transporting V0 subunit D2 (Atpase d2) [11, 24]. Ethanol treatment also upregulated the bone resorption genes Trap (Acp5) and cathepsin K (Ctsk) [25] in a time-dependent manner (Figure 2A).

**Table 1:** Sequences of primers used in real-time PCR.

**Gene**	**Forward primer**	**Reverse primer**
*β-actin*	5′-AGC CAT GTA CGT AGC CAT CC-3′	5′-CTC TCA GCA GTG GTG GTG AA-3′
*TRAP*	5′-TCC TGG CTC AAA AAG CAG TT-3′	5′-ACA TAG CCC ACA CCG TTC TC-3′
*CTSK*	5′-CTT CCA ATA CGT GCA GCA GA-3′	5′-TCT TCA GGG CTT TCT CGT TC-3′
*c-Fos*	5′-CCA GTC AAG AGC ATC AGC AA-3′	5′-AAG TAG TGC AGC CCG GAG TA-3′
*NFATc1*	5′-CAG CTG CCG TCG CAC TCT GGT C-3′	5′-CCC GGC TGC CTT CCG TCT CAT A-3′
*V-ATPd2*	5′-AAG CCT TTG TTT GAC GCT GT-3′	5′-TTC GAT GCC TCT GTG AGA TG-3′
*DC-STAMP*	5′-CTT GCA ACC TAA GGG CAA AG-3′	5′-TCA ACA GCT CTG TCG TGA CC-3′
ALDH1A1	5′- AACACAGGTTGGCAAGTTAATCA -3′	5′- TGCGACACAACATTGGCCTT-3′

The disulfiram was later performed to investigate its role on these master genes which acted during the process of osteoclastogenesis. As the results showed, disulfiram significantly decreased the expression of these genes which up-regulated by ethanol (Figure 2B).

These data revealed that disulfiram dramatically suppressed osteoclast-related genes expression.

### Disulfiram abrogated the expression of NFATc1, which was up-regulated by ethanol

NFATc1 has been demonstrated to be a master regulator of RANKL-induced OC differentiation [26, 27], which is modulated via RANKL-induced downstream pathways. Binding of RANKL to the RANK receptor results in the recruitment of TNF receptor-associated factor 6 [28], which is involved in activating downstream signaling pathways, such as the NF-κB, AKT, JNK, p38, and ERK pathways [27, 29–31]. Additionally, OC differentiation critically depends on c-Fos expression in progenitor cells [32], and osteoporosis did not occur in the absence of c-Jun [33]. Because our data showed that osteoclastogenesis was facilitated by ethanol, we further investigated whether ethanol targeted these factors. The data revealed that ethanol promoted the PI3K-AKT, MAPKs and NF-κB signaling pathways which play important roles during osteoclastogenesis (Supplementary Figure 1).

The role of ethanol on these OC-related signaling pathway final contributed to the master factor NFATc1, c-Fos and c-Jun we indicated previously. The results (Figure 2C) showed that NFATc1, c-Fos, and c-Jun were induced on days 1 and 3 of RANKL stimulation; however, co-treatment with ethanol markedly increased the induction of NFATc1 protein expression in response to RANKL but did not affect c-Fos and c-Jun. In addition, a luciferase assay was used to confirm that ethanol stimulated the expression of NFATc1 by affecting its transcriptional activity (Figure 2E). Our data indicate that ethanol increased osteoclastogenesis via NFATc1.

Combining previous data, we thus hypothesis whether disulfiram inhibited ethanol-induced osteoporosis via NFATc1. As the data showed (Figure 2F and 2G), disulfiram decreased the expression of NFATc1 in a dose-dependent manner.

These data indicate that ethanol promoted RANKL-induced osteoclastogenesis via increasing the expression of NFATc1 while the treatment of disulfiram down-regulating the expression of NFATc1. However, the deep mechanism of disulfiram on NFATc1 was still unclear.

### Disulfiram inhibits ethanol-induced osteoclastogenesis via ALDH1A1

**Figure 3:**
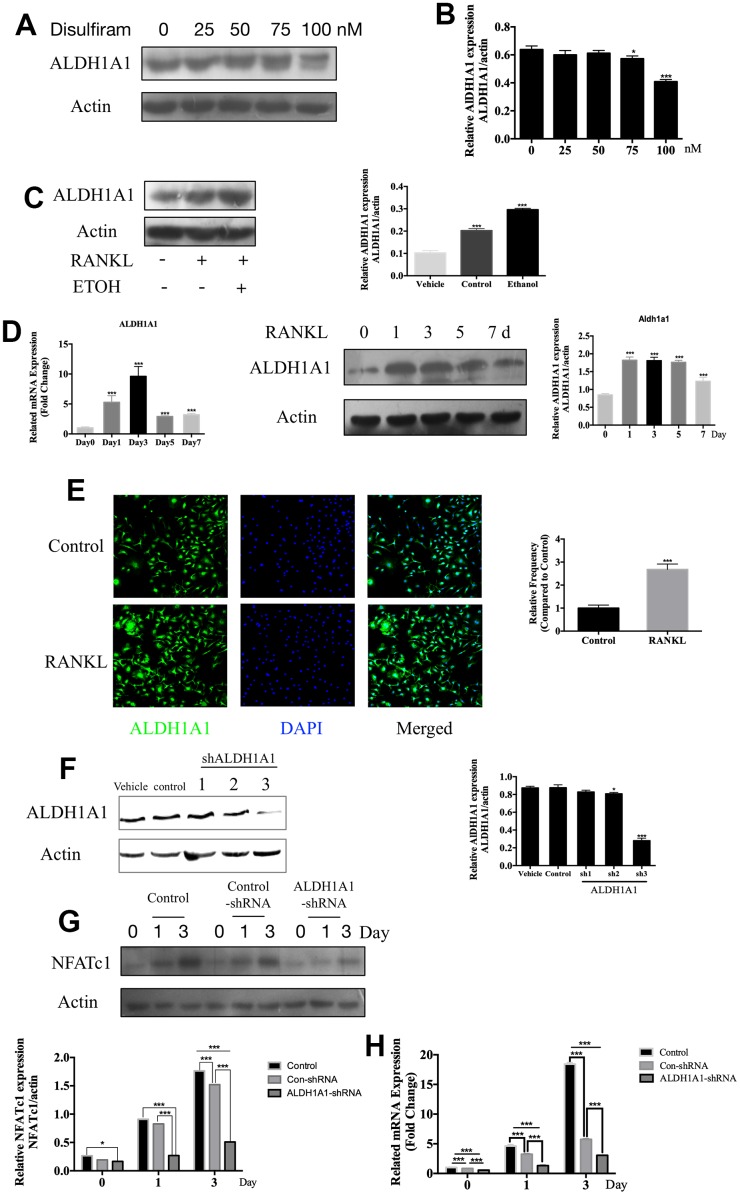
**Disulfiram inhibits ethanol-induced osteoclastogenesis via ALDH1A1.** (**A**) Disulfiram inhibited the expression ALDH1A1 in a dose-dependent manner. (**B**) Relative expression of ALDH1A1 was determined by densitometric analysis of each band and expressed as a ratio to that of β-actin using ImageJ software. (**C**) Ethanol increased the expression of ALDH1A1. Total cellular proteins were extracted from BMM-derived OCs treated with 100 ng/mL RANKL, with or without ethanol, for 3 days. Relative expression of ALDH1A1 was determined by densitometric analysis of each band and expressed as a ratio to that of β-actin using ImageJ software. (**D**) qPCR was used to measure the relative levels of ALDH1A1 expression, normalized to those of β-actin, at different times of osteoclastogenesis (*n* = 3). Values are the mean ± SD. *p < 0.05, **p < 0.01, ***p < 0.001. Western blotting was used to measure the relative levels of ALDH1A1 expression at different times of osteoclastogenesis. Total cellular proteins were extracted from BMM-derived OCs treated with RANKL for 0, 1, 3, 5, and 7 days. (**E**) Representative immunofluorescence images of ALDH1A1 from BMMs treated with or without RANKL, showing that RANKL increased ALDH1A1 in the cytoplasm and the nucleus. Nuclei were counterstained with DAPI (blue). (**F**) Efficiency of ALDH1A1 silencing was determined by immunoblot analysis. Relative expression of ALDH1A1 was determined by densitometric analysis of each band and expressed as a ratio to that of β-actin using ImageJ software. (**G**) Silencing of ALDH1A1 decreased the expression of NFATc1. Total cellular proteins were extracted from RAW 264.7 cells and RAW 264.7 cells transfected with Lenti-ALDH1A1 or Lenti-NC, after co-treatment with RANKL for 0, 1, and 3 days. (**H**) qPCR was used to measure the relative expression levels of ALDH1A1, normalized to those of β-actin (*n* = 3). Values are the mean ± SD. *p < 0.05, **p < 0.01, ***p < 0.001.

Disulfiram has long been evaluated as an inhibitor which targets at ALDH1A1 [23, 34, 35], and accumulated studies had revealed the vital position of ALDH1A1 in alcohol related diseases [16, 22, 36, 37]. The data showed 100nM disulfiram significantly inhibited the expression of ALDH1A1 (Figure 3A), confirming the inhibition of disulfiram on ALDH1A1. Subsequently, the treatment of ethanol dramatically increases the expression of ALDH1A1 (Figure 3C), we thus further explored the effect of ALDH1A1 during the process of osteoclastogenesis,

The role of ALDH1A1 during different periods of osteoclastogenesis was later examined. The qPCR data indicated that *Aldh1a1* expression was dramatically increased by stimulation with RANKL, particularly on day 3; western blotting showed similar results (Figure 3D). Additionally, immunofluorescence staining of ALDH1A1 revealed a greater increase in ALDH1A1 under stimulation with RANKL than in the unstimulated group, either in the cytoplasm or nucleus (Figure 3E).

Our previous data demonstrated the inhibition of disulfiram on the expression of NFATc1, as disulfiram was an inhibitor of ALDH1A1, we next silenced ALDH1A1 via transfection of Lenti-ALDH1A1 into RAW 264.7 cells. The efficiency of ALDH1A1 silencing was determined by western blotting (Figure 3F). Silencing of ALDH1A1 decreased the expression of NFATc1 compared to in the control group and con-shRNA group in a time-dependent manner (Figure 3G and 3H). Combining previous data, downregulating and silencing of ALDH1A1 significantly inhibited the expression of NFATc1, ALDH1A1 was thus considered as an upstream factor of NFATc1, promoted RANKL-induced OC differentiation.

In summary, our data indicated the important role of ALDH1A1 in the process of osteoclastogenesis and the ALDH1A1 was an up-stream factor of NFATc1, ALDH1A1-NFATc1 axis may played important role during the process of osteoclastogenesis.

### Disulfiram rescued Alcohol-Induced bone loss and reduction in bone mechanical strength in mice

**Figure 4:**
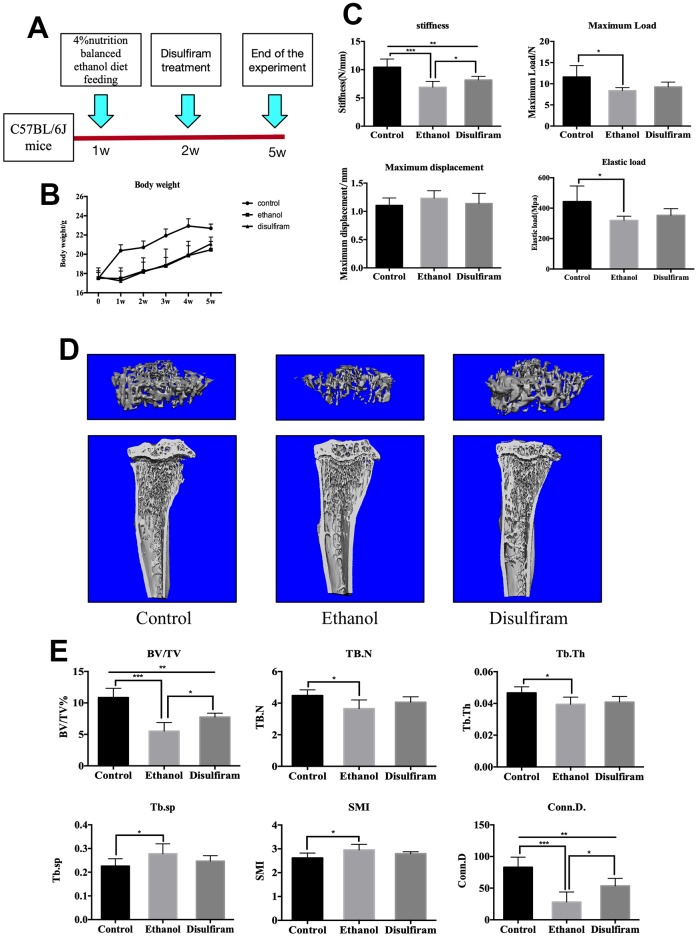
**Disulfiram rescues alcohol-induced bone loss and reduction in bone mechanical strength in mice.** (**A**) Schematic representation of the animal model. (**B**) Body weights of mice. (**C**) Mechanical properties (ultimate stiffness, maximum load, elastic load, and maximum displacement) of femurs. (**D**) Representative 3D micro-CT reconstruction images of mice in the control, ethanol, and disulfiram groups. (**E**) Quantitative analyses of morphometric parameters of the bone volume to tissue volume (BV/TV, %), trabecular number (Tb.N), trabecular thickness (Tb.Th), trabecular spacing (Tb.Sp), trabecular connectivity density (Conn.D), and the structure model index (SMI) of the regions of interest (*n* = 6). Bar graphs are presented as the mean ± SD. *p < 0.05, **p < 0.01, and ***p < 0.001 compared to the respective controls.

Confirming the effect of ALDH1A1 in vitro, We further investigated the role of ALDH1A1 in ethanol-induced osteoporosis *in vivo*, an animal model was developed, as schematically presented in Figure 4A. Body weight measurement during the experimental period indicated that alcohol consumption led to a lower body weight in mice compared to in the control group, the weight of treatment group was resembling to the ethanol group (Figure 4B). Osteoporosis is associated with an increased risk of fracture, leading to deterioration of mechanical properties of the bone; thus, we further explored the relationship between alcohol consumption and the mechanical properties of bone. As shown in Figure 4C, alcohol consumption decreased bone stiffness, maximum load, and elastic load but did not increased the maximum displacement of femurs, while treatment with disulfiram prevented the effects on stiffness only. Next, tibias were analyzed by micro-CT, and the data showed that trabecular bone mass in the ethanol group was markedly reduced compared to in the control group, which was confirmed by 3D reconstruction (Figure 4D). However, the data obtained for the disulfiram-treated group revealed a dramatic rescuing effect. Quantitative bone parameters showed significant reductions in the BV/TV ratio, trabecular number, and connectivity density and marked increases in trabecular spacing and the structure model index (a measure of rod- and plate-like geometries of the trabecular bone) after chronic alcohol consumption. Treatment with disulfiram reduced the negative impact of ethanol on the bone and improved the bone morphometric parameters on BV/TV and Conn.D (Figure 4E).

### Disulfiram protected against alcohol-induced bone loss via ALDH1A1

**Figure 5:**
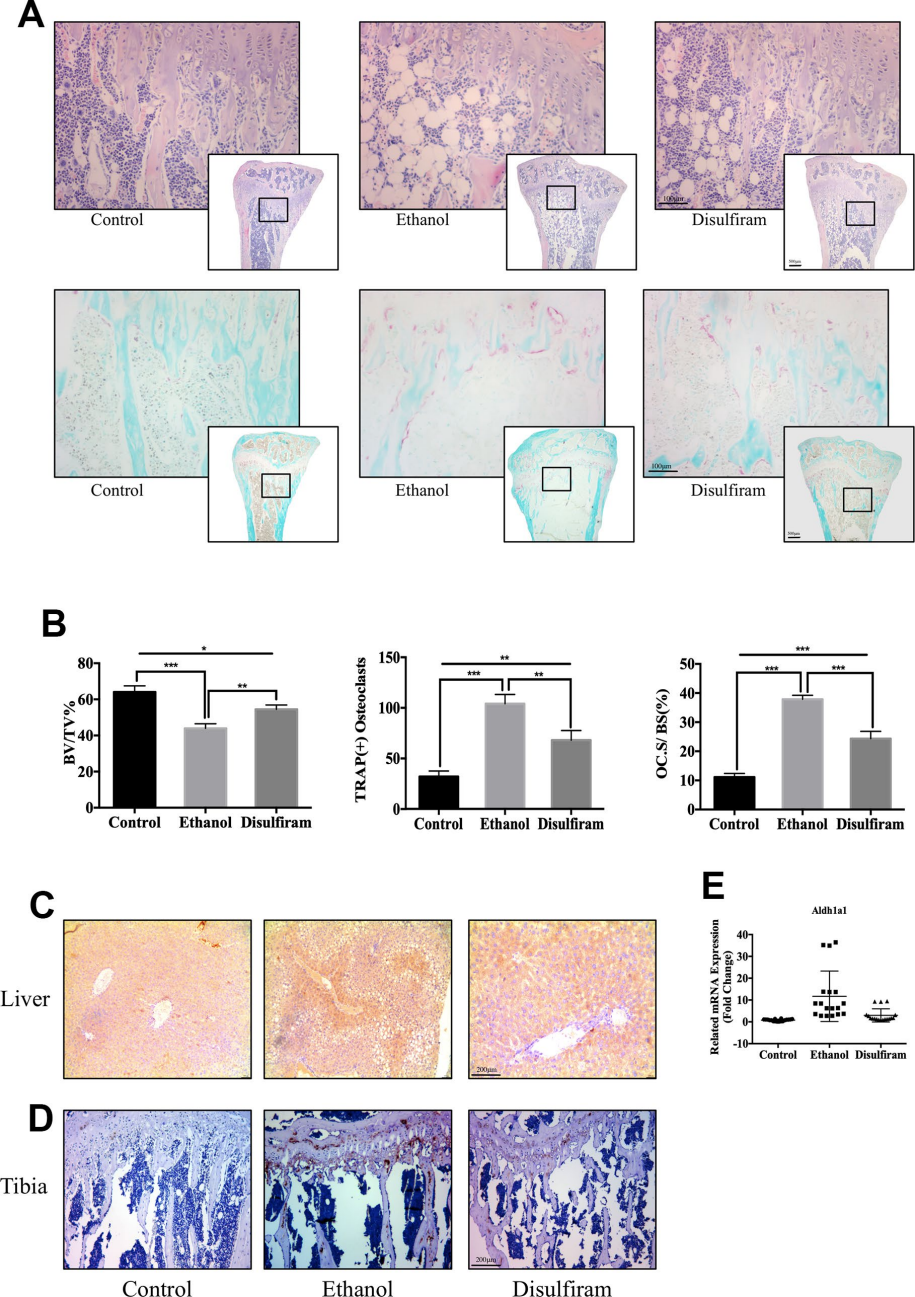
**Disulfiram protected against Alcohol-Induced bone loss via ALDH1A1.** (**A**) Sections of tibias were stained with H&E and TRAP. (**B**) Data of quantitative analyses of histomorphometric bone parameters of the bone volume to tissue volume (BV/TV), number of TRAP-positive OCs, and percentage of the OC surface to bone surface (Oc.S/BS, %) (*n* = 6). (**C**) Immunohistochemical staining of ALDH1A1 expression in the liver. (**D**) Immunohistochemical staining of ALDH1A1 expression in the tibia. (**E**) qPCR was conducted to measure the relative levels of gene expression, normalized to those of β-actin, in each group of mice (*n* = 6). Bar graphs are presented as the mean ± SD. *p < 0.05, **p < 0.01, and ***p < 0.001 compared to the respective controls.

Histomorphometric analysis of the tibia was conducted to assess the protective effect of disulfiram against ethanol-induced bone loss. Consistent with the micro-CT results, H&E staining further confirmed the beneficial effect of disulfiram on the bone volume compared to the alcohol consumption group (Figure 5A and 5B). The reduced OC activity on the bone surface and significant reduction in the total number of TRAP-positive OCs in the bone (Figure 5A and 5B) also demonstrated the dramatic protective effect of disulfiram against alcohol-induced osteoporosis.

To examine the expression of ALDH1A1 *in vivo*, immunohistochemical staining for ALDH1A1 was performed in the liver and tibia. The results showed marked ALDH1A1 positivity in the liver and bone trabecula in the ethanol group compared to in the control group (Figure 5C and 5D). In contrast, disulfiram treatment decreased the expression of ALDH1A1 in both the liver and bone trabecula. In addition, qPCR indicated that ethanol increased the expression of *Aldh1a1* in each group, while disulfiram protected against this increase (Figure 5E). Combined with the *in vitro* data, these findings demonstrate that alcohol consumption induced the expression of ALDH1A1, while disulfiram protected against it, and thus targeting ALDH1A1 may be a potential method of therapy for osteoporosis.

Our data indicate that an inhibitor of ALDH1A1, disulfiram, exerted strong protective effects against alcohol-induced bone loss.

## DISCUSSION

Since its discovery, alcohol has become the most popular drink in the world and has also caused many problems. Chronic alcohol consumption results in various diseases, including osteoporosis as a severe consequence, affecting tens of thousands of patients. A treatment method is urgently needed for osteoporosis, and we thus examined the underlying mechanism of alcohol-induced osteoporosis to identify potential therapeutic strategies. In this study, we investigated the inhibition of disulfiram on ethanol-induced osteoclastogenesis via decreasing the expression of NFATc1, we also found that the role of ALDH1A1 during the process of osteoclastogenesis, and the silence of ALDH1A1 lead to the down-regulation of NFATc1, the master factor of osteoclastogenesis, these data indicated ALDH1A1-NFATc1 axis a potential therapy target of osteoporosis.

Osteoporosis is a multifactorial disease that occurs because of dysregulation of bone metabolism or homeostasis. Disruptions in bone homeostasis shifts the balance in favor of increasing OC formation and function, leading to excessive bone destruction. Because OCs represent the only known cell type capable of resorbing the bone matrix, excessive OC bone resorption leads to osteoporosis and tumor-associated bone destruction [11], while impaired OC formation and/or bone resorption result in osteoporosis [12]. We first explored the effect of ethanol on osteoclastogenesis. Our data demonstrated that ethanol significantly promoted RANKL-induced OC formation and bone resorption *in vitro*, and OC-related genes were also upregulated by ethanol. Disulfiram, an anti-alcoholism drug, has long been clinically used for the treatment of chronic alcoholism, and its inhibition on RANKL-induced osteoclastogenesis was previous explored [15]. Otherwise, previous studies also indicated disulfiram an effective inhibitor which dramatically suppressed MAPK, NF-κB signaling pathways, and also abrogated the expression of V-ATPases, decreasing resorption effect of osteoclasts [38–40]. However, the effect of disulfiram on alcohol-related osteoporosis is still unclear. Our data revealed the treatment of disulfiram abrogated ethanol-induced OC formation and bone resorption, otherwise, the OC-related genes were also inhibited by disulfiram.

NFATc1 was considered as the master regulator of RANKL-induced OC differentiation, and NFATc1-deficient BMMs fail to differentiate into OC [27]. Our data demonstrated that ethanol significantly facilitated the expression of NFATc1. Our study also revealed that disulfiram dramatically suppressed the expression of NFATc1, the inhibiting effect of disulfiram on ethanol-induced osteoclastogenesis was thus contributed to NFATc1. However, the deep mechanism is still unclear.

Ethanol is first metabolized into acetaldehyde through several enzymatic and nonenzymatic mechanisms [16]. Alcohol dehydrogenases and ALDHs are the principal enzymes responsible for ethanol metabolism in humans [41]. In the human ALDH family, mitochondrial ALDH2 and cytosolic ALDH1A1 are the major isoforms responsible for the metabolism of acetaldehyde. To date, ALDH1A1 has been associated with alcohol-induced flushing in Caucasians as well as alcohol sensitivity and dependence [22, 36], along with its functions in the detoxification of acetaldehyde, the first metabolite of ethanol oxidation [37]. Accumulated studies had long evaluated disulfiram an inhibitor which targets at ALDH1A1 [23, 34, 35]. We thus further explored the role of ALDH1A1 during the process of osteoclastogenesis and the relationship between ALDH1A1 and NFATc1. Our data revealed the inhibition of 100nM disulfiram on ALDH1A1 in vitro. The expression of ALDH1A1 was increased by ethanol compared to in the control group. Further observations revealed that ALDH1A1 was upregulated during RANKL-induced osteoclastogenesis, particularly on day 3. This indicates that ALDH1A1 has a vital function in osteoclastogenesis. Additional researches indicated that the silence of ALDH1A1 decreased the expression of NFATc1. Those data revealing that ALDH1A1 is an upstream factor of NFATc1 and can promote RANKL-induced OC differentiation.

Based on this promising *in vitro* data, we further investigated the role of ALDH1A1 *in vivo* in a Lieber–DeCarli liquid diet-fed C57 mice. Our results indicate that ethanol consumption highly increased the expression of ALDH1A1 in the liver and tibia of mice, while treatment with disulfiram rescued ethanol-induced damage of bone loss and reductions in its mechanical strength.

Taken together, our study confirmed that ethanol actively promoted RANKL-induced osteoclastogenesis and disulfiram was an effective therapy agent for ethanol-induced osteoporosis. The deep mechanism mainly contributed to the target factor of disulfiram-ALDH1A1. The major ALDH isoform-ALDH1A1 is dramatically increased by ethanol and inhibited by disulfiram, it is responsible for the metabolism of acetaldehyde, and it also played a vital role during the process of osteoclastogenesis. Silencing of ALDH1A1 led to a decrease of NFATc1, the master regulator of RANKL-induced OC differentiation. Moreover, disulfiram also decreased ethanol-induced osteoporosis *in vivo*, making ALDH1A1 a potential target for future treatment of ethanol-induced osteoporosis and osteoclast-osteoporosis.

## MATERIALS AND METHODS

### Media and reagents

Alpha modified minimal essential medium (a-MEM), Dulbecco’s modified eagle medium (DMEM), fetal bovine serum (FBS), and penicillin/streptomycin were purchased from Trace (Sydney, Australia). Disulfiram was purchased from MCE (New Jersey, USA). RANKL and Macrophage Colony-Stimulating Factor (M-CSF) were purchased from R&D Systems (Minneapolis, MN, USA). Specific antibodies against Phosphatidylinositol 3-kinase (PI3K) (#4249), protein kinase B (also known as AKT; #9272), phosphorylated (p)-AKT (#4060), extracellular regulated protein kinases (also known as ERK #4695), p-ERK (#4370), p38 (#8690), p-p38 (#4511), p65 (#8242), p-p65 (#3033), c-Jun N-terminal kinase (also known as JNK #9252, p-JNK (#9255), c-Fos (#2250), c-Jun (#9165), inhibitor of NF-κB (IκBα; #4814), nuclear factor of activated T cells c1 (NFATc1; #8032), and β-actin (#3700) were obtained from Cell Signaling Technology (Danvers, MA, USA). The ALDH1A1 antibody (#15910-1-AP) was obtained from Proteintech Group (Rosemont, IL, USA).

### Cell culture

Primary bone marrow-derived macrophages (BMMs) were extracted from the femoral and tibial bone marrows of 6-week-old male C57BL/6 mice and maintained in α-MEM supplemented with 10% FBS, 1% penicillin/streptomycin, and 30 ng/mL M-CSF (complete α-MEM) in a humidified incubator of 37°C and 5% CO_2_. Raw 264.7 cells were maintained in α-MEM supplemented with 10% FBS and 1% penicillin/ streptomycin in a humidified incubator of 37°C and 5% CO_2_. 293T cells were maintained in DMEM supplemented with 10% FBS and 1% penicillin/ streptomycin in a humidified incubator of 37°C and 5% CO_2_.

### TRAP staining

To measure tartrate-resistant acid phosphatase (TRAP) activity, M-CSF-dependent BMMs were seeded in triplicate into a 96-well plate at a density of 8 × 10^3^ cells/well in complete α-MEM supplemented with 25 ng/mL M-CSF and 100 ng/mL RANKL and incubated in the absence or presence of ethanol at concentrations of 12.5, 25, and 50 mM. At the end of the experiment, the cells were washed twice with PBS, fixed by 4% paraformaldehyde (PFA) for 30 min, and then stained for TRAP activity. Mature OCs were quantified by counting the number of multinucleated (>3 nuclei) TRAP-positive cells in a representative area in each of three replicate samples.

### Pit formation assay

Pro-osteoclasts were produced by seeding BMMs (5 × 10^3^ cells/well) into 96-well culture plates and stimulated with 100 ng/mL RANKL and 25 ng/M-CSF for 5 days until small osteoclastic cells began to form. The pro-osteoclasts were seeded onto bovine bone discs and stimulated with RANKL and M-CSF without or with the indicated concentrations of ethanol. The media was changed every other day; after 5 days of culture, the cells were cleaned by mechanical agitation and sonication. Bone resorption pits were examined by using a scanning electron microscope (Field Environmental Instruments, Inc., Hillsboro, OR, USA). The area of the resorption pits was measured with Image-Pro Plus software (Media Cybernetics, Rockville, MD, USA).

### RNA isolation and quantitative PCR

Quantitative PCR (qPCR) was used to evaluate the expression of osteoclastic genes during RANKL-induced OC formation. M-CSF-dependent BMMs were seeded into a 6-well plate and stimulated with RANKL in the absence or presence of 50 mM ethanol for 1, 3, 5, or 7 days. Total RNA was isolated from each well by using the RNAiso Plus total RNA extraction reagent (Takara Bio, Shiga, Japan) according to the manufacturer’s protocol. First-strand cDNA was synthesized using the PrimeScript RT cDNA synthesis kit (Takara Bio) and 1 μg of the extracted RNA template. qPCR was then carried out using SYBR Green real-time PCR master mix (Takara Bio) and the following cycling parameters: 40 cycles at 94°C for 20 s, 60°C for 20 s, and 72°C for 30 s. The results were normalized to the expression levels of β-actin as an internal housekeeping gene. The primer sets used are listed in Table 1.

### Western blot analysis

Cellular proteins were treated under the indicated conditions in an incubator maintained at 37°C with 5% CO_2_. At the end of the experiment, the cells were washed twice with PBS and collected using cell lysis buffer. After incubating the cells in lysis buffer for 30 min on ice, the samples were centrifuged at 15,000 ×*g* for 10 min at 4°C. The supernatant containing protein was collected, and protein concentration was measuring with a bicinchoninic acid protein assay kit. After normalizing the protein concentration, the sample was mixed with sodium dodecyl sulfate-sampling buffer, followed by boiling at 95°C for 5 min. Protein (40 μg) were loaded onto 10–12% SDS-PAGE gels and subsequently transferred to 0.22-μm polyvinylidene fluoride membranes. The membrane was incubated in blocking buffer for 1 h. The membranes were washed with Tris-buffered saline containing Tween 20 and then incubated with appropriate horseradish peroxidase-conjugated secondary antibodies at room temperature for 1 h. The blocked membranes were then incubated with primary antibodies overnight at 4°C, and then incubated with the secondary antibody for 1 h. Finally, the bands were detected via ECL Western Blotting Substrate and visualized with a Bio-Rad ChemiDoc system (Hercules, CA, USA).

### Immunofluorescence microscopy

BMMs were seeded onto bovine cortical bone slices in a 12-well plate at a density of 3 × 10^5^ cells/well and incubated for 24 h. The glass coverslips with BMMs were washed three times with PBS, and then the cells were fixed with 4% PFA for 30 min and permeabilized with Triton X-100 for 10 min at room temperature. After blocking with 5% bovine serum albumin for 30 min, the BMMs were incubated with primary antibodies against ALDH1A1 (1:500) and p65 (1:500) overnight. BMMs were then incubated with Alexa Fluor 530-conjugated IgG secondary antibodies for 1 h and stained with 4′,6-diamidino-2-phenylindole (DAPI) for 5 min. Images were analyzed under a Nikon Eclipse Ti microscope (Tokyo, Japan) and Olympus FV1200 microscope (Tokyo, Japan).

### Luciferase gene reporter assay

RAW 264.7 cells stably expressing the 3κB-Luc-SV40 luciferase reporter gene construct, as previously described, [44] cells were plated at a density of 1.5 × 10^5^ cells/well in 48-well plates and pretreated with 12.5, 25, and 50 mM ethanol for 1 h, followed by stimulation with 100 ng/mL RANKL for a further 6 h. Unstimulated cells were used as a negative control, and cells treated with RANKL without ethanol were used as a positive control. After incubation, the cells were harvested, and cell lysates were used to determine the luciferase activity using a luciferase assay system (Promega, Madison, WI, USA) according to the manufacturer’s instructions.

### Lentivirus production

ALDH1A1-silencing plasmids (pPLK/GFP+Puro-ALDH1A1) were purchased from the Public Protein/ Plasmid Library (Jiangsu, China). The short hairpin ALDH1A1 mouse-specific sequences were as follows: 5′-CGGATTTAGGAGGCTGCATAA-3′, 5′-CGCAATG AAGATATCTCAGAA-3′, and 5′-GCTCATGTTCATT TGGAAGAT-3′. The ALDH1A1-silencing plasmids were transfected into 293T cells using Lipofectamine 3000 according to the manufacturer’s instructions. After approximately 4 h, the medium was replaced with fresh normal medium, and the supernatants were collected after additional incubation for 48 and 72 h.

### Transfection

ALDH1A1 was silenced via transfection of cells at a confluence of 30–50% with Lenti-ALDH1A1 (obtained as previously described); Lenti-NC was used as a control. The cells were selected using 7.5 μg/mL puromycin. After the fluorescence intensity reached 70%, transfection efficacies were measured by western blotting.

### Establishment of an alcoholic osteoporosis animal model

All animal care and experimental procedures were approved by the Zhejiang University Institutional Animal Care and Use Committee (No. 14666). Six-week-old C57BL/6J male mice were randomly divided into three groups (*n* = 6 per group), including a control group, ethanol-fed group, and disulfiram-treated group, and housed in a temperature-controlled room under a 12-h light/dark cycle. The ethanol-fed and disulfiram-treated groups were fed 4% (v/v) ethanol in the Lieber–DeCarli liquid diet (TROPHIC, Nantong, China) over a 5-week period (Figure 7A), while the control group was fed a control diet (TROPHIC). After consuming the liquid diet for 1 week, the mice in the disulfiram-treated group were intragastrically administered with disulfiram (30 mg/kg) every other day dissolved in a 20% sodium carboxymethyl cellulose solution; the other groups were intragastrically administered with 20% sodium carboxymethyl cellulose solution. Four weeks later, the mice were sacrificed, and their femurs and tibias were excised and processed for mechanical testing, micro-CT analysis, and histological staining. During the process of experiment, all mice were still alive.

### Bone mechanical testing

Mechanical properties of the femurs and L_5_ bones were evaluated using a material testing machine (Instron 4465, Norwood, MA, USA). A destructive three-point bend test was performed at the right femur midshaft at a compression rate of 5 mm/min and stabilized with a static preload of 0.5 N. Force versus displacement data were collected at 100 Hz, and stiffness (N/mm) was evaluated as previously described [45].

### Micro-CT scanning

Tibias were fixed in 4% PFA for 2 days and then analyzed using a high-resolution micro-CT scan at an isometric resolution of 18 μm with the X-ray energy set to 80 kV and 100 μA. Cone-beam reconstruction software (SkyScan, Aartselaar, Belgium) was used to reconstruct 3D images. A nummular region of interest (3 × 3 × 1 mm), centered around the midline suture, was used to analyze the osteolysis-related index. The ratio of the bone volume to tissue volume (BV/TV, %) and the number and area of pores within the region of interest were measured using CT analyzer software (SkyScan).

### Histological analysis and immunohistochemistry

After fixation in 10% formaldehyde for 3 days, tibias were decalcified in 10% EDTA (PH7.4) for 21 days, dehydrated, and embedded in paraffin. Paraffin blocks contain tibias were sliced in a coronal for further TRAP staining and *He*matoxylin-Eosin staining (H&E staining) and were analyzed under a high-quality microscope. The number of TRAP-positive multinucleated OCs, osteoclasts per bone surface (OCs/BS), and the percentage of BV/TV (%) were analyzed using ImagePro Plus 6.0 software. Sections (Tibias and Livers) were immunostained with anti-rabbit ALDH1A1 (1:200, proteintech) antibodies for 12h, followed by detection using a HRP-conjugated secondary antibody and 3,3′-Diaminoben- zidine (DAB) (Gene Tech, Shanghai, China) to measure the expression of proteins, and were analyzed under a high-quality microscope.

### Statistical analysis

All data presented are representative of at least three independent experiments and are expressed as the mean ± SD. Statistical differences were assessed by a Student's *t*-test, one-way ANOVA, and two-way ANOVA, followed by a least-significant difference test, using SPSS 19.0 software (SPSS, Inc., Chicago, IL, USA). A value of *p* < 0.05 was considered statistically significant.

## Supplementary Material

Supplementary Materials

Supplementary Figure 1
