# Supplementary material for: Disulfiram suppressed ethanol promoted RANKL-induced osteoclastogenesis in vitro and ethanol-induced osteoporosis in vivo via ALDH1A1-NFATc1 axis
Source: Aging (Albany NY). 2019 Oct 8;11(19):8103–19. doi: 10.18632/aging.102279 (PMC6814600; doi:10.18632/aging.102279)
Supplement: Supplementary Materials [file aging-11-102279-s001.pdf]

SUPPLEMENTARY MATERIALS

Ethanol stimulates RANKL-induced activation of NF-κB, MAPK, and PI3K/AKT pathways

Because of the important roles of the NF-κB, MAPK, and PI3K/AKT pathways in modulating RANKL-induced osteoclastogenesis [31, 42, 43], we further investigated the effects of ethanol on these pathways. BMMs were cultured in serum-free medium with or without ethanol (50 mM) for 1 h. The cells were treated with RANKL, with or without ethanol, for 0, 5, 10, 20, 30, and 60 min. The data indicated that ethanol activated the NF-κB pathway (Figure 2A). Phosphorylation of p65 was significantly increased by ethanol at each time point, and ethanol prevented the degradation of IκBα, an upstream regulator of NF-κB, which regulates the nuclear translocation of NF-κB p65. Immunofluorescence staining of p65 also revealed that ethanol promoted translocation of p65 from the cytoplasm to the nucleus (Figure 2C).

Our data also demonstrated that ethanol activated the PI3K/AKT and MAPK signaling pathways (Figure 2E). RANKL stimulation resulted in rapid phosphorylation of AKT and all three components of the MAPK cascade (ERK, p38, and JNK) (Figure 2E). Phosphorylation of these key members results in activation of the downstream cascades required for OC formation.

Graph abstract

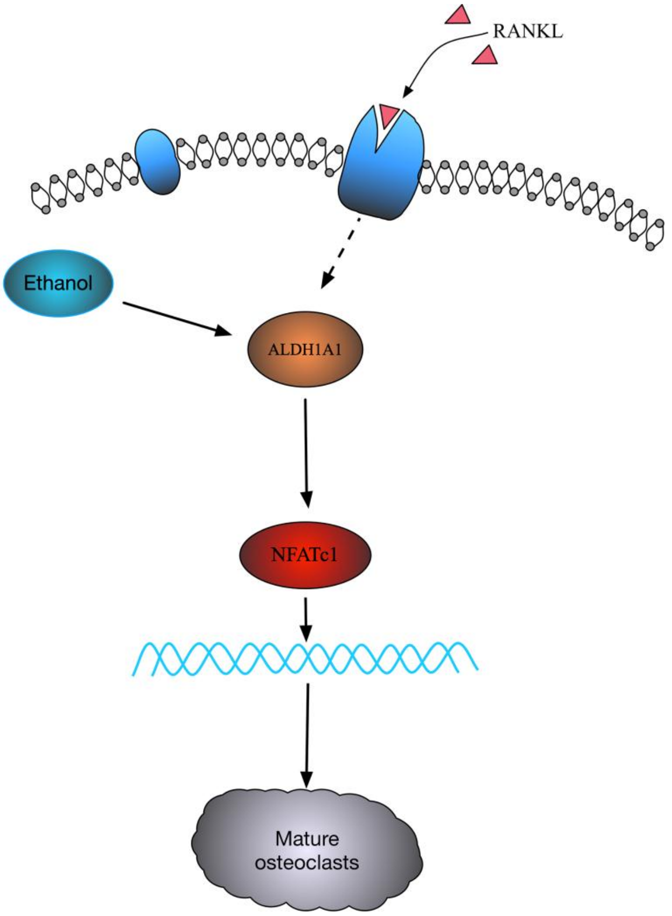

The graph abstract demonstrates the role of ALDH1A1 during the ethanol promoting RANKL-induced osteoclastogenesis
