# Supplementary material for: Disulfiram suppressed ethanol promoted RANKL-induced osteoclastogenesis in vitro and ethanol-induced osteoporosis in vivo via ALDH1A1-NFATc1 axis
Source: Aging (Albany NY). 2019 Oct 8;11(19):8103–19. doi: 10.18632/aging.102279 (PMC6814600; doi:10.18632/aging.102279)
Supplement: Supplementary Figure 1 [file aging-11-102279-s002.pdf]

SUPPLEMENTARY FIGURE

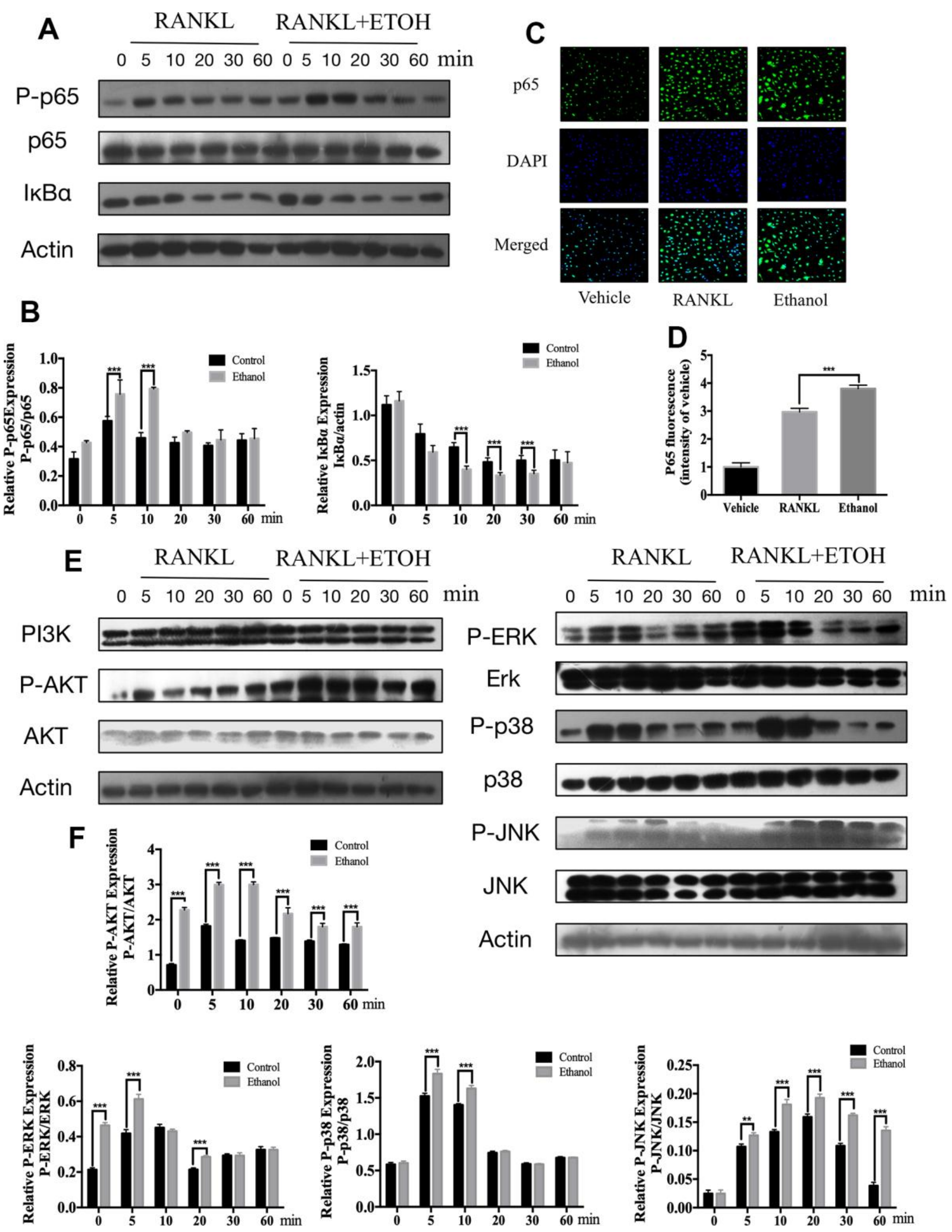

Supplementary Figure 1. Ethanol stimulates RANKL-induced activation of the NF-κB, MAPK, and PI3K/AKT pathways, ultimately increasing NFATc1 expression. Total cellular proteins were extracted from BMMs pretreated with 50 mM ethanol for 1 h and then stimulated with 100 ng/mL RANKL for the indicated times. (**A**) Ethanol promoted IκBα degradation and p65 phosphorylation. (**B**) Expression levels of p-p65, relative to those of total p65, and IκBα, relative to those of β-actin, were determined using ImageJ software. (**C**) Representative immunofluorescence images of p65 localization (green) in BMMs treated with ethanol and stimulated with RANKL, showing that ethanol promoted p65 nuclear translocation. Nuclei were counterstained with DAPI (blue). (**D**) Relative frequency of p65 under each experimental condition was quantified using ImageJ software (*n* = 3). (**E**) Ethanol increased the phosphorylation of AKT, ERK, p38, and JNK. (**F**) Relative changes in the phosphorylation status of AKT, ERK, p38, and JNK were determined by densitometric analysis of each phosphorylated band and expressed as a ratio to its total protein counterpart using ImageJ software. Data are the mean ± SD. *$p < 0.05$, **$p < 0.01$, and ***$p < 0.001$ compared to the respective controls.
